# Automated detection and growth tracking of 3D bio-printed organoid clusters using optical coherence tomography with deep convolutional neural networks

**DOI:** 10.3389/fbioe.2023.1133090

**Published:** 2023-04-12

**Authors:** Di Bao, Ling Wang, Xiaofei Zhou, Shanshan Yang, Kangxin He, Mingen Xu

**Affiliations:** ^1^ School of Automation, Hangzhou Dianzi University, Hangzhou, China; ^2^ Key Laboratory of Medical Information and 3D Bioprinting of Zhejiang Province, Hangzhou, China

**Keywords:** organoid, optical coherence tomography, tracking, convolutional neural network, deep learning

## Abstract

Organoids are advancing the development of accurate prediction of drug efficacy and toxicity *in vitro*. These advancements are attributed to the ability of organoids to recapitulate key structural and functional features of organs and parent tumor. Specifically, organoids are self-organized assembly with a multi-scale structure of 30–800 μm, which exacerbates the difficulty of non-destructive three-dimensional (3D) imaging, tracking and classification analysis for organoid clusters by traditional microscopy techniques. Here, we devise a 3D imaging, segmentation and analysis method based on Optical coherence tomography (OCT) technology and deep convolutional neural networks (CNNs) for printed organoid clusters (Organoid Printing and optical coherence tomography-based analysis, OPO). The results demonstrate that the organoid scale influences the segmentation effect of the neural network. The multi-scale information-guided optimized EGO-Net we designed achieves the best results, especially showing better recognition workout for the biologically significant organoid with diameter ≥50 μm than other neural networks. Moreover, OPO achieves to reconstruct the multiscale structure of organoid clusters within printed microbeads and calibrate the printing errors by segmenting the printed microbeads edges. Overall, the classification, tracking and quantitative analysis based on image reveal that the growth process of organoid undergoes morphological changes such as volume growth, cavity creation and fusion, and quantitative calculation of the volume demonstrates that the growth rate of organoid is associated with the initial scale. The new method we proposed enable the study of growth, structural evolution and heterogeneity for the organoid cluster, which is valuable for drug screening and tumor drug sensitivity detection based on organoids.

## 1 Introduction

Organoids are 3D self-organized assemblies of stem cell or neoplastic cell derived from patient tumor (Shahbazi et al., 2019) with more similarities to the source organs with respect to their morphological and functional characteristics (Tuveson & Clevers, 2019), providing a new platform for precise drug screening and tumor drug sensitivity detection *in vitro* (Takebe & Wells, 2019). In recent years, an increasing variety of cancer organoid have been successfully established, including but not limited to liver and bile duct (Broutier et al., 2017), bladder (Lee et al., 2018; Mullenders et al., 2019), esophagus (Li et al., 2018), lung (Sachs et al., 2019), intestine (Boehnke et al., 2016), and stomach (Seidlitz et al., 2019). The organoid undergoes the process of self-organogenesis when dispersed in the culture matrix and has a multi-scale histomorphology of 30–800 μm (closely related to functional and tumor heterogeneity). Hence, the traditional techniques such as fluorescence microscopy and Laser Scanning Confocal Microscopy usually lack the ability of achieving long-term, non-destructive, 3D imaging and classification analysis of organoid clusters. In addition, traditional biochemical detection techniques such as detection of adenosine triphosphate (ATP) are subjected to the diffusion of culture media and substances, making it difficult to distinguish the function states among different scales of organoids. For instance, cancer organoids with diameter ≥50 μm are reported to more accurately predict the effects of antitumor drugs (Nuciforo et al., 2018; Chung et al., 2022; de Medeiros et al., 2022; Kang et al., 2022). Therefore, there is an urgent need to establish a label-free and non-invasive imaging and analysis method that can perform 3D imaging, classification, tracking and functional analysis of organoid clusters.

Optical coherence tomography (OCT) is an emerging biomedical imaging modality that enables high-resolution, label-free, non-destructive and real-time 3D imaging of biological tissues and has been widely used in ophthalmology (Ngo et al., 2020) and dermatology (Pfister et al., 2019). With the millimeter-level penetration depth and micrometer-level resolution, OCT can characterize the internal structures of organoids with high resolution. In addition, the label-free advantage of OCT allows for longitudinal monitoring of organoids for the number and morphology changes. For instance, Deloria et al. (2021) used ultra-high resolution 3D OCT to observe the internal structure of human placenta-derived trophoblast organoids. Gil et al. (2021) used OCT to track the volumetric growth of patient-derived intestinal cancer organoids, which employed k-means clustering to segment the organoids in OCT images and achieved quantitative tracking of individual organoid volumes with diameter larger than 100 μm. However, the current study (Oldenburg et al., 2015; Gil et al., 2021) both analyzed the heterogeneity of drug responses in mature organoids through traditional image processing combined with OCT, without considering the morphology changes in organoid development. The in-time reveal of tissue morphology during organoid growth and the evaluation of appropriate developmental time scales would be beneficial to guide organoid culturing (Yavitt et al., 2021). Assessing morphological changes during organoid growth requires improved accuracy of organoid quantification, especially for organoids with diameters around 50 μm (Gjorevski et al., 2016; Arora et al., 2017). However, poor contrast, unclear boundaries, the presence of noise, and the small size of the organoid target in OCT images poses a challenge in accurate organoids identification for current methods.

Recently, convolutional neural networks (CNNs) have emerged as a powerful tool in image classification (Cai et al., 2020), object detection (Xiao et al., 2020) and image segmentation (Hesamian et al., 2019). These networks exploit an encoder-decoder architecture. In detail, the encoder enables it to obtain low-resolution feature maps, then the decoder is utilized to project the low-resolution feature maps to high-resolution feature maps to achieve pixel-based classification. In organoid image analysis, CNNs have achieved automatic segmentation of organoids in 2D microscope images and confocal images, and the corresponding segmentation results outperform traditional image processing and machine learning methods (Kassis et al., 2019; MacDonald et al., 2020; Bian et al., 2021; Abdul et al., 2022). Also, CNNs has similarly shown good results in many OCT image processing tasks. For example, CNNs have been employed for segmentation of choroidal vessel (Liu et al., 2019), capillary (Mekonnen et al., 2021), retinal layer boundaries (Shah et al., 2018) and skin (Kepp et al., 2019). Despite CNNs show considerable promise in OCT image analysis, their application in OCT images is still in the early stages (Pekala et al., 2019; Badar et al., 2020).

To better detect and tracking morphological changes of printed patient-derived tumor organoid clusters, we propose a 3D imaging, segmentation and analysis method based on OCT technology with CNNs (Organoid printing and OCT-based analysis, OPO). Specifically, we exploit a designed inverted OCT system to perform automated, high-throughput imaging of organoids arising from patient-derived cancerous tissues such as liver, colon, and stomach. Moreover, two neural network joint organoid segmentation and classification algorithms are correspondingly designed to identify multi-scale organoids in OCT images with high accuracy. Based on the method of organoid mass alignment to achieve the tracking of individual organoids and the tracking analysis of multi-scale structures of organoid clusters within microbeads, we analyze the morphological and number changes of different individual organoids of the same species and different species of organoid clusters, providing a new method to study the growth, structural evolution and heterogeneity of organoid clusters.

## 2 Methods

### 2.1 Patient-derived cancer organoids preparation and culture

Multiple patient-derived cancer organoids (PCOs) were prepared by our proposed dot extrusion bioprinting (Wei et al., 2022), and a total of 52 samples of microbead organoid clusters of different patient sources were obtained, including three types of cancer organoids: liver, stomach, and intestine. Cells from patient-derived cancerous tissues were first digested with 0.25% trypsin, then suspended in base medium (DMEM/F12 with 10% FBS and 1% penicillin-streptomycin), and mixed with Matrigel in a 1:2 ratio to prepare the bioink for printing. Briefly, dot extrusion printing is a method of bioprinting using an extrusion printhead operating in intermittent mode to generate microbeads onto the target substrate surface by transient contact. The droplet-shaped microbeads will be deposited on the substrate due to the surface tension between the hydrogel and the substrate. The bioink was loaded into the extrusion printhead and kept at 37°C. Subsequently, the cell-laden bioink was printed in 96 multi-well plates using a micro-extrusion nozzle under pre-designed G-code commands with pneumatic pressure set to 100 kPa and dispensing time set to 1,000 ms. The multi-well plates were incubated at 37°C for 2–3 min to solidify the mixture and then invert to ensure free growth of PCOs suspended in the 3D environment of the Matrigel. The mixture was left to solidify for at least 20 min, then base medium supplemented with Wnt3a medium (1:1 ratio) was added to each well and the medium was renewed every 2 days during long-term incubation.

### 2.2 Image acquisition and preprocessing

**FIGURE 1:**
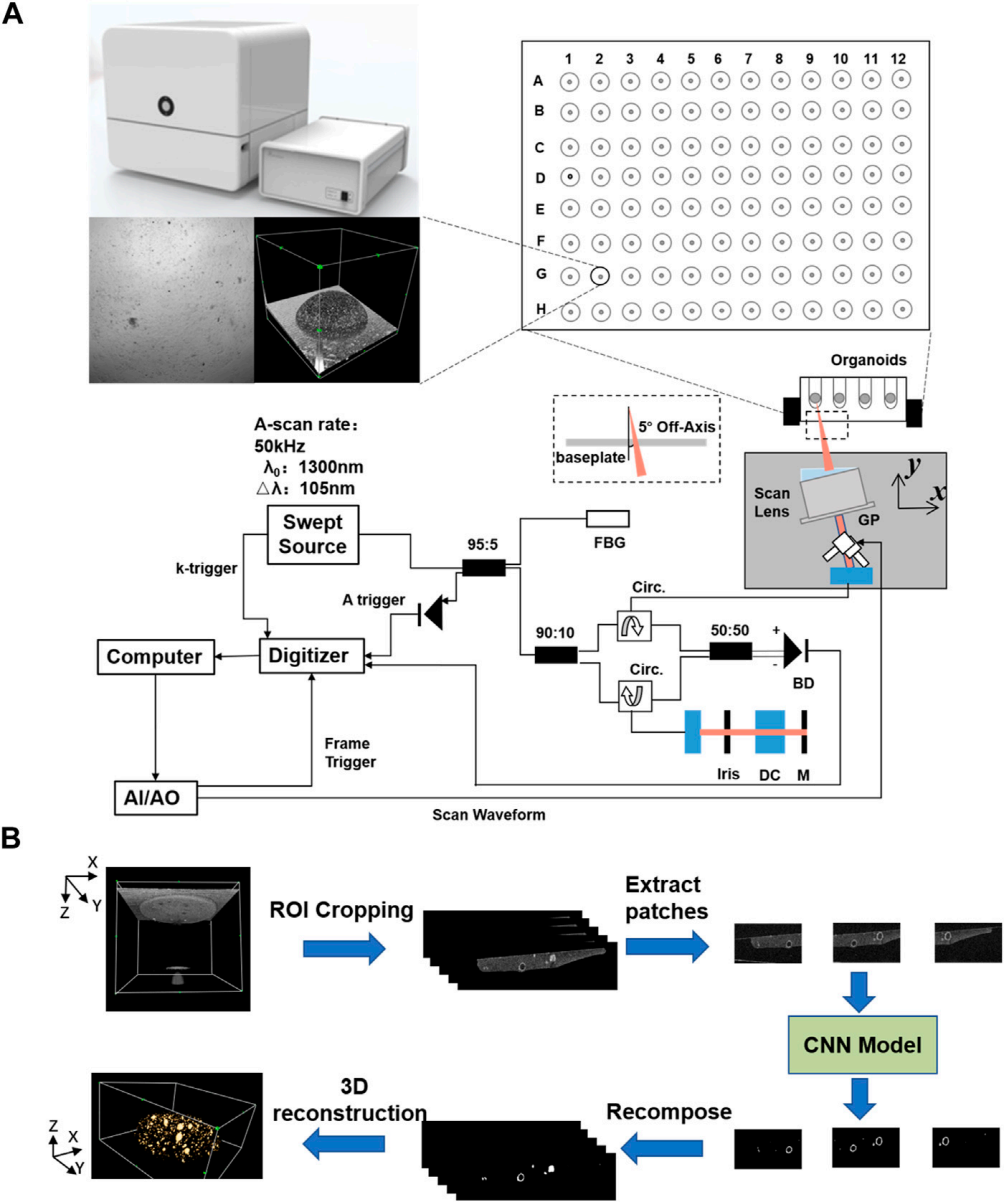
Schematic diagram of the SS-OCT system and the data processing flowchart for organoid imaging and segmentation. **(A)** Schematic diagram of the SS-OCT system for organoid imaging, **(B)** The data processing flowchart for organoid segmentation.

We applied the SS-OCT system developed by Regenovo (Figure 1, Bio-Architect@ Tomography, Regenovo, China) to acquire 3D images of the organoid. The system has a central wavelength of 1,310 nm, an axial resolution of 7.6 μm and a lateral resolution of 15 μm. The OCT images were resized in the axial direction to yield isometric voxel spacing. It was noteworthy that the data used in this paper were observed using inverted imaging and tilted by 5–10° with the aim of reducing noise. For organoid clusters within printed microbeads, OCT data acquisition and data storage took no more than 30 s in total, and each OCT imaging monitoring did not exceed 1 h to reduce the impact of imaging monitoring on organoid growth. We started OCT imaging monitoring which last for 7 days when we observed most of the organoids sprouting under 4x microscope, and the time interval of each data acquisition was 24 h. Thus, a total of 364 sets of 3D OCT data were obtained for 52 samples of printed organoid clusters. The volume size of each group of 3D OCT images was 715(z) × 800(x) × 800(y) voxel, and the total field of view was 3.58 mm(z) × 4 mm(x) × 4 mm (y).

We selected 3-4 well-developed organoid samples for each of the three tumor types and chose different time points to cover the entire culture cycle. The final dataset contains 10 samples with a total of 24 OCT data, which is about 1/5 of all samples. Due to the high workload of supervised machine learning algorithms that require real annotated data for training and testing, it is not feasible to segment the entire OCT data manually. Therefore, each 3D body data in the 24 OCT data was annotated every four frames, and 3729 B-Scan images were finally obtained. We adjusted the contrast of the images and denoised the images by filtering so that the organoid could be more easily identified. The ground truth images are manually annotated by experts under the guidance of bright-field images, and the data were divided into two parts and annotated from the orthogonal direction. We first compared the bright-field images for determining the position of the organoid in the OCT images. Then the ground truth baseline is established by morphological characteristics of the organoid, signal intensity, and continuity of the organoid (adjacent B-scan images). Considering that the organoids below 32 μm are not biologically significant and their labeling errors are easy to occur, we set the lower bound of organoid size to 32 μm (Gil et al., 2021). In this work, the software tool ITK Snap was used.

### 2.3 Designing convolutional neural network models to segment organoids

**FIGURE 2:**
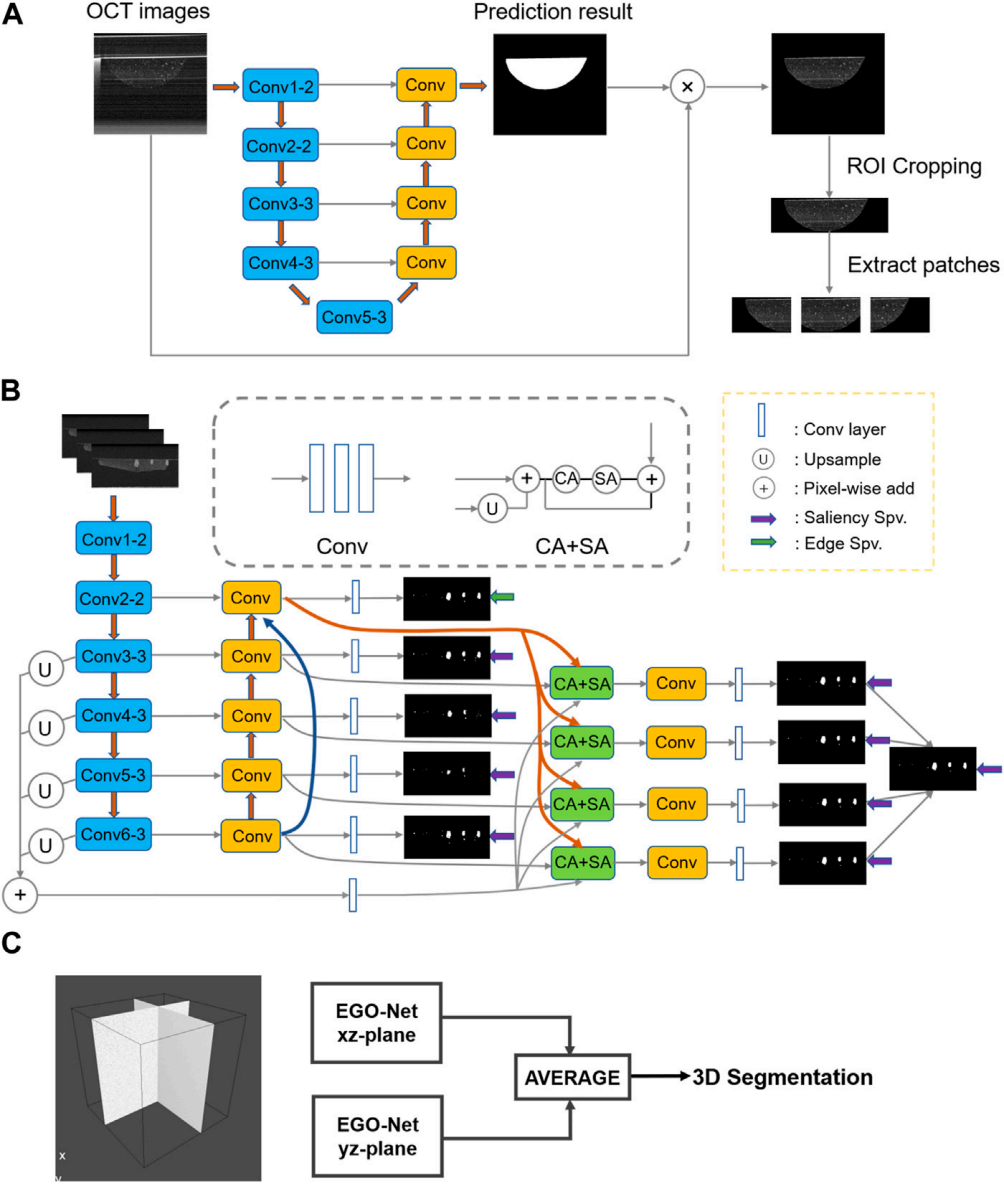
The network structure or method used in the entire segmentation process. **(A)** Determine the VGG-Unet structure for the region of interest microbeads. **(B)** Architecture of the proposed EGO-Net. (The left side shows the encoder and decoder combination with U-shaped structure. The encoder receives the image as input and generates multi-level and multi-resolution feature representations, while fusing the features after Conv2-2 to obtain multi-scale features. The decoder then receives the feature representation from the encoder to generate the corresponding predicted output, and uses the output corresponding to Conv2-2 to generate edge features. The segmentation results are obtained on the right side by fusing the multi-scale features, predicted outputs and edge features and decoding them accordingly.) **(C)** The final 3D segmented image obtained by averaging results of two orthogonal direction.

During 3D printing, the deviations in microbead positions and manual manipulation caused by different collectors may lead to the organoid appearance at different locations in the OCT images. In this paper, a simple neural network, VGG-Unet (Simonyan & Zisserman, 2014; Ronneberger et al., 2015), was first used to extract the printed microbead matrix region of interest (Figure 2A). Then the printing errors (volume and position deviations) were calibrated and the interference signals were removed, to accurately reconstruct the multiscale structure of the organoid clusters within the printed microbeads.

After obtaining OCT images [240 (z) *800 (x) pixels] containing only Matrigel and organoid clusters, we performed preprocessing such as cropping and grayscale transformation to resolve the grayscale and non-uniform contrast of OCT images in different organoids. Then dividing a B-scan image containing organoid clusters into three 240(z)*400(x) pixel image blocks would achieved preserving the organoid integrity while reducing the size of the input image, which enabled it improve the segmentation efficiency and accuracy of the neural network. Then, we exploited four method, U-Net, U^2^-Net (Qin et al., 2020), EG-Net (Zhao et al., 2019) and nnU-net (Isensee et al., 2018) based CNNs, for multi-scale organoid segmentation of the pre-processed OCT images. In detail, the first three CNNs are 2D neural networks, and nnU-net was the 3D neural network with the best segmentation performance so far (Antonelli et al., 2021).

The morphological sprouting of the organoid generally occurs when the diameter reaches 50 μm. In order to better evaluate the morphological changes during the growth of the organoid, we designed EGO-Net to improve the segmentation accuracy of the morphological sprouting state organoid, as shown in Figure 2B. Since the lower level has a small receptive field, only local information can be obtained. In order to accurately segment organoids, high-level semantic or positional information is also required. When the information returns from the high level to the low level, the high level information is gradually diluted, so we fuse the feature maps obtained from each convolutional block of the down sampled part of the introduced baseline EG-Net to obtain the multi-scale features. The multi-scale features are then fused with the features of the side path outputs so that both high-level and low-level information is obtained for each side path output. Channel Attention (CA) and Spatial Attention (SA) were utilized after fusing the edge features to make the network more focused on the organoids to be segmented and to suppress irrelevant information (Woo et al., 2018). Since this network (Figure 2B) was still a 2D neural network, the correlation information of different directions of 3D OCT imaging was easily lost, which led to problems such as deformation and discontinuity in the 3D reconstruction of the segmented organoid. To this end, a method combining unidirectional continuous 3-frame OCT image input (Li et al., 2017) and averaging two predictions in the orthogonal direction (Prasoon et al., 2013; Pfister et al., 2019) was proposed (Figure 2C). This method took into account the information correlation of different directions of 3D OCT imaging and used orthogonal continuous input to form information complementarity, which enabled effectively improve the problem of unidirectional 2D neural network segmentation while avoiding the segmentation efficiency problem that exists in 3D neural networks.

We trained the network using a hybrid loss function defined as $L=L_{bce}+L_{iou}.$ $L_{bce}=-\sum_{i=1}^{n}\hat{y_{i}}\log y_{i}+\left(1-\hat{y_{i}}\right)\log\left(1-y_{i}\right)$ and $L_{iou}=1-\frac{\sum_{i=1}^{n}y_{i}\hat{y_{i}}}{\sum_{i=1}^{n}\left(\hat{y_{i}}+y_{i}-y_{i}\hat{y_{i}}\right)}$ denote the BCE loss function (de Boer et al., 2005) and the IoU loss function (Mattyus et al., 2017), respectively, where $\hat{y_{i}}\in \left[0,1\right]$ is the ground truth label of the pixel and $y_{i}\in \left[0,1\right]$ is the predicted probability of being organoid. We set six-fold cross-validation with 60 epochs of training per cross-validation and used data augmentation such as rotation and horizontal flipping during the training process. An adaptive moment estimation solver was used to optimize the network with a learning rate of 5 × 10^−5^ and momentum of 0.90. This process was implemented with Python3.7 based on a Pytorch backend using a single NVIDIA Quadro RTX 4000.

### 2.4 Organoid tracking and quantitative analysis

The individual tracking of organoid within microbeads, individual organoid volume, overall number and volume, and clustering analysis of volume can be achieved based on the segmentation results of the CNN model proposed in this paper. The organoid volume is calculated only for the solid part and not for the whole contained in the outer contour. A three-point localization method was used for OCT imaging of the organoid clusters in the culture well plate to ensure the consistent position of each data acquisition as much as possible. Moreover, this paper proposed a combined ICP alignment and center-of-mass pairing approach to achieve time-dependent tracking of individual organoid within a cluster. Specifically, we first identified and labeled the organoids within the microbeads based on the segmentation results of the CNN model, then used a simplified point cloud based iterative closest point (ICP) algorithm to align the organoid segmentation datasets at different time points. Next, we extracted the mass center of each organoid from the aligned datasets, and created a pairing table by matching the mass center of the closest organoid in the aligned datasets at two adjacent time points. In turn, we created a pairing table for multiple time points for organoid tracking, which enabled the tracking of a single organoid. The results of individual organoid tracking were used to demonstrate the change of organoid number and morphology over time.

### 2.5 Evaluation metrics

Four different metrics were used to evaluate the accuracy of the segmentation. Dice and Jaccard were used to measuring the similarity between the predicted and ground truth, which are denoted as:

$$Dice=\frac{2TP}{2TP+FN+FP},$$ (1)

$$Jaccard=\frac{TP}{TP+FN+FP},$$ (2)

where true positives (TP) are the number of pixels for which the model correctly predicts the positive class, false positives (FP) are the number of pixels for which the model incorrectly predicts the positive class, and false negatives (FN) are the number of pixels for which the model incorrectly predicts the negative class.

Dice and Jaccard are more concerned with the overlap region between predicted and ground truth, and the size of the segmented region also has an impact on the results. Therefore, we selected the precision and sensitivity for indicating the classification of positive samples. The precision indicates the probability of correct prediction in the samples predicted as organoid, and a higher precision indicates fewer cases of predicting the background as organoid. It is defined as:

$$Precision=\frac{TP}{TP+FP}.$$ (3)

Sensitivity indicates the probability of correct prediction in samples that are actually organoid, and higher sensitivity indicates fewer cases of predicting organoid as background. It is denoted as:

$$Sensitivity=\frac{TP}{TP+FN}.$$ (4)

## 3 Results

### 3.1 Quantitative analysis of segmentation performance

Previous studies reveal that morphogenesis in the organoid would occur at the diameter of 50 μm (Gjorevski et al., 2016; Arora et al., 2017), which was also observed in our study. Therefore, we compared our proposed method with other single neural networks according to different classifications of the equivalent diameter of the organoid, and the metrics of all models for test set segmentation are shown in Table 1. It should be pointed out that other neural networks do not use a series of optimized processing methods, for example, VGG-Unet to extract the printed microbead matrix region, and orthogonal continuous input prediction to solve the problems such as deformation and dislocation in the 3D reconstruction of the organoid after segmentation. From the table, it can be seen that the segmentation performance of our proposed method is better than other neural networks, and obtained the highest scores in Dice, Jaccard, Precision and Sensitivity. When the diameter of the organoid is between 32 and 50 μm, our method can achieve more than 80% segmentation accuracy. When the organoid diameter was between 50 and 90 μm, the segmentation performance of each model improved, with our method improving between 3% and 6% compared to nnUnet, U2net, and EG-Net, and 9.6% compared to U-net. When the organoid diameter is larger than 90 μm, our method improves 4.8% compared to EG-Net, the best performing of other single neural network, and 8.4% compared to U-net. Our method has the best segmentation performance for different organoid diameters, indicating that our method can accurately identify the organoid and effectively improve the segmentation accuracy.

**TABLE 1:** Comparison between the proposed segmentation method and other single neural network.

	Diameter	U-net	nnUnet	U^2^Net	EG-net	Our method
Dice	50 > d ≥ 32 μm	0.680	0.710	0.753	0.758	0.801
	90 > d ≥ 50 μm	0.743	0.785	0.786	0.809	0.839
	d ≥ 90 μm	0.820	0.828	0.850	0.856	0.904
Jaccard	50 > d ≥ 32 μm	0.546	0.552	0.635	0.635	0.683
	90 > d ≥ 50 μm	0.619	0.624	0.683	0.701	0.737
	d ≥ 90 μm	0.707	0.718	0.763	0.767	0.832
Precision	50 > d ≥ 32 μm	0.722	0.681	0.777	0.781	0.824
	90 > d ≥ 50 μm	0.808	0.759	0.821	0.843	0.854
	d ≥ 90 μm	0.892	0.861	0.899	0.901	0.902
Sensitivity	50 > d ≥ 32 μm	0.700	0.715	0.779	0.780	0.802
	90 > d ≥ 50 μm	0.733	0.657	0.791	0.809	0.844
	d ≥ 90 μm	0.765	0.853	0.837	0.829	0.921

**FIGURE 3:**
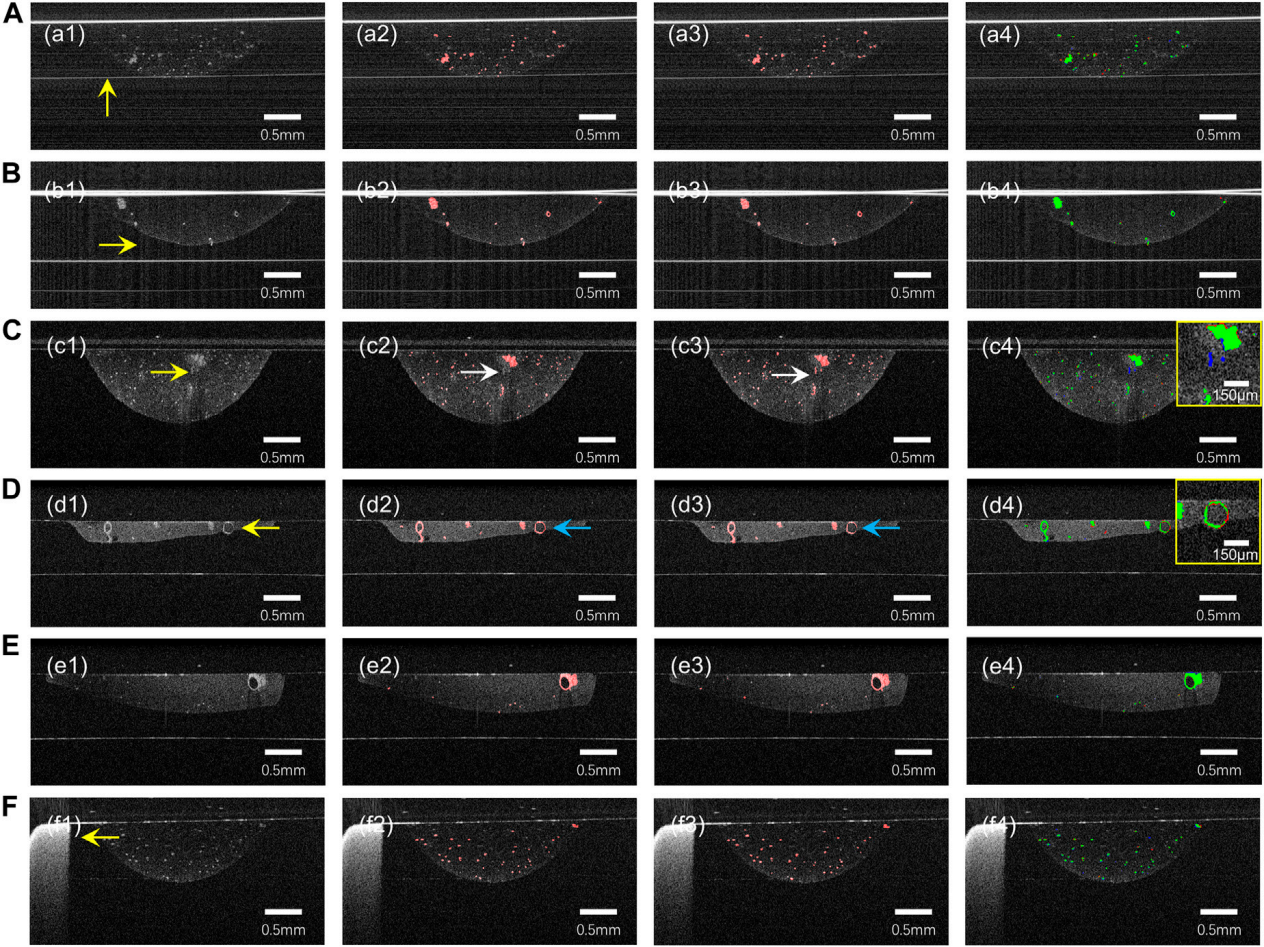
The prediction results of our model for some challenging cases of B-Scan. **(A)** autocorrelation noise; **(B)** strong reflection noise; **(C,D)** low contrast and blurred boundaries; **(E)** different density and morphology of organoid. **(F)** Well plates. (a1)–(f1) are OCT images, (a2)–(f2) are ground truth, (a3)–(f3) are neural network prediction results, and (a4)–(f4) are fusion results (green) of ground truth (red) and prediction results (blue). The yellow arrows indicate possible problems in the OCT images. The white arrow in row 3 points out that Matrigel was misclassified as an organoid. The blue arrow in row 4 points out that the organoid was not successfully segmented.

As can be seen from Figure 3, our model provides satisfactory segmentation for the problems that exist in OCT imaging in the normal culture environment of the organoid clusters such as the difference in growth density, morphological differences (Figure 3E), preparation process limitations (Figure 3F), and the strong reflection noise, autocorrelation noise (Figures 3A, B), and low contrast (Figures 3C, D). While it is prone to generate errors only in cases where expert recognition is also difficult, such as organoid with diameter less than 50 μm (Figure 3A), strong signal of Matrigel compared to surrounding signal (as shown in Figure 3C), or close signal intensity of organoid and Matrigel (as shown by arrow in Figure 3D).

**FIGURE 4:**
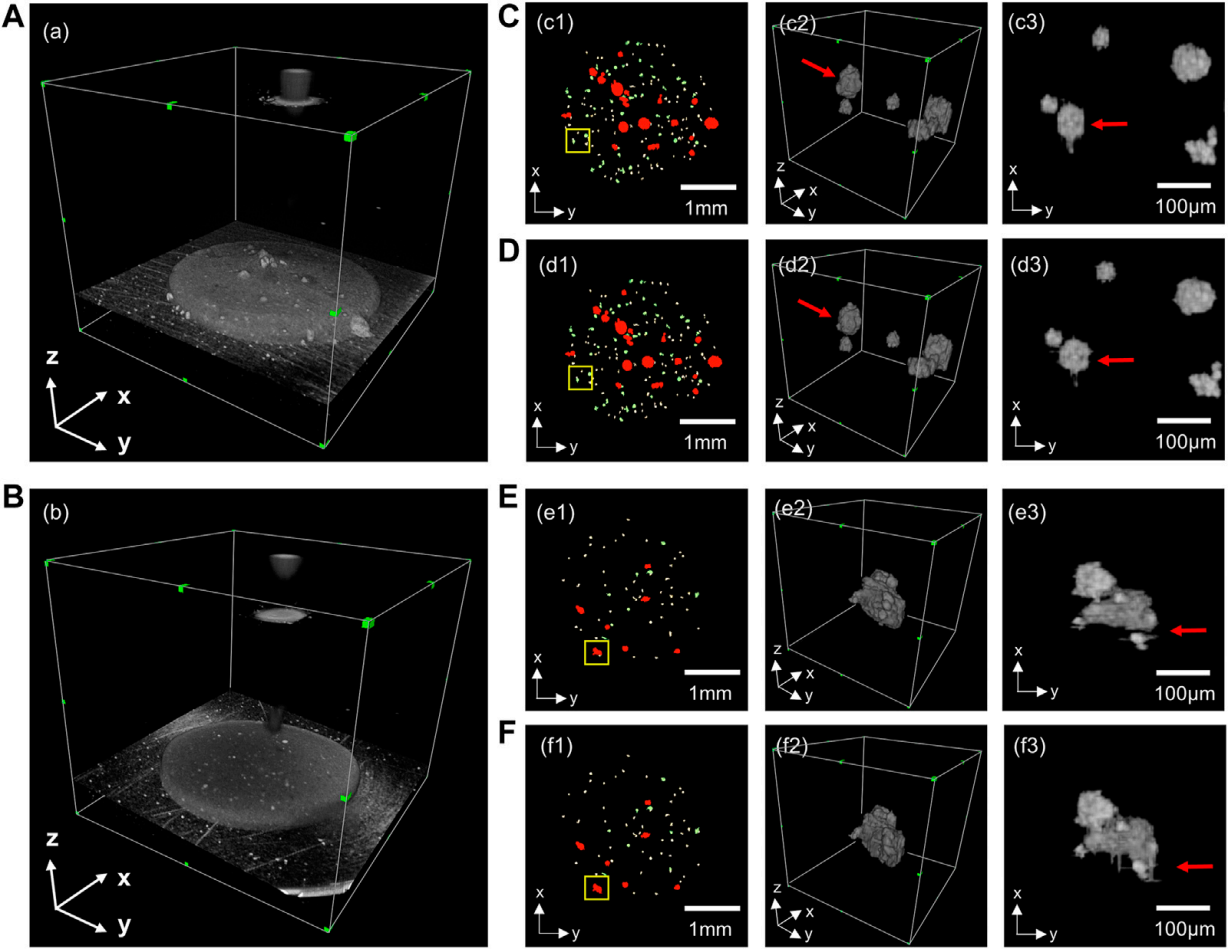
Comparison of the 3D segmentation results of unidirectional prediction and orthogonal continuous input prediction. **(A,B)** are two sets of 3D OCT examples; **(C,E)** are 3D segmentations with unidirectional prediction. **(D,F)** are 3D segmentations with orthogonal continuous input prediction. (c1–f1) are top views of 3D reconstructions of organoids segmentation; (c2–f2) are enlarged 3D reconstructions of the yellow boxes in (c1–f1); (c3–f3) are top views corresponding of (c2–f2).

We used the orthogonal continuous input prediction method to improve the error-prone problem of unidirectional prediction of 2D neural network, and compared the effect of unidirectional prediction and orthogonal continuous input prediction, as shown in Figure 4. Due to the lack of information in the third dimension, the 2D neural network unidirectional prediction leads to problems such as deformation, missing edges (Figure 4C3) and discontinuity (Figure 4E3) in some of the organoids, which cannot accurately demonstrate the morphological changes in the organoids, and thus the quantitative analysis of multi-scale organoid clusters would also produce errors. The orthogonal continuous input prediction method effectively improves the impact due to the missing information in the third dimension. As can be seen from Figure 4D3, the orthogonal continuous input prediction method used in this paper segmented the shape of the organoid more rounded and complete; as can be seen from Figure 4F3, the method in this paper could reconnect the defective organoid predicted unidirectionally by the 2D neural network into a whole, which improves the accuracy of organoid identification.

### 3.2 Analysis of organoid growth based on segmentation results

**FIGURE 5:**
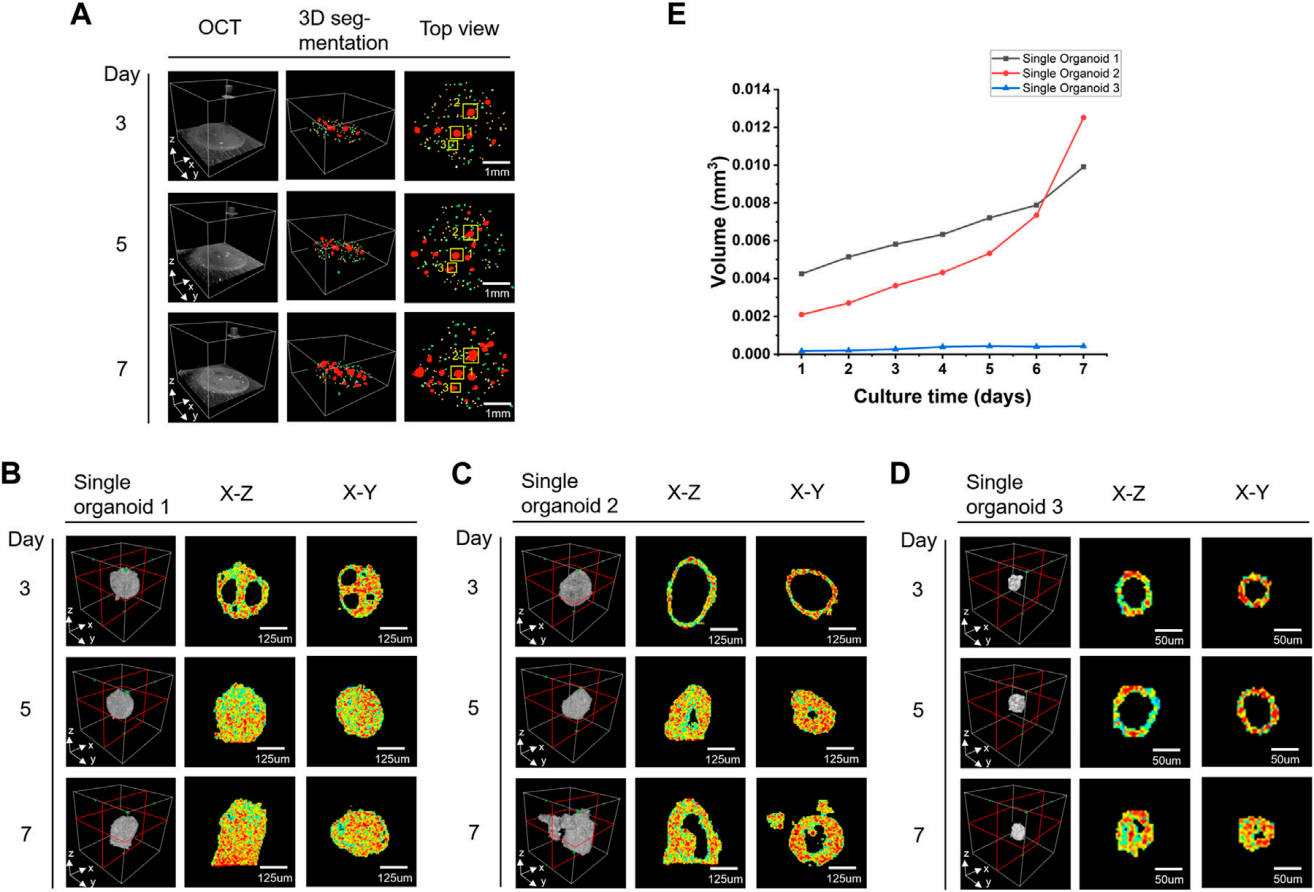
Shape and size changes of colon cancer organoids during the monitoring period. **(A)** Colon cancer organoids at different time points. From left to right are 3D OCT images, 3D segmentation results and the top views. Different diameters of organoids are marked with different colors (yellow:50 μm > d ≥ 32μm, green: 90 μm > d ≥ 50μm, red: d ≥ 90 μm). **(B–D)** Enlarged views of the three organoids marked as 1, 2, and 3 by the yellow boxes in **(A)**. **(E)** The volume growth analysis of three organoids in **(B–D)** over 7 days.

Figure 5A shows the growth changes of the colon cancer organoids during the monitoring period, where the different sizes of the organoids are presented in different colors. Three individual organoids were selected for display (Figures 5B–D) and volume analysis (Figure 5E). Different organoids showed different morphologies in the same well of the same patient. The organoid growth around 150 μm for initial diameter may exhibit almost consistent morphology in appearance but experience significant differences in internal structure, such as the gradual disappearance of the cavity of organoid 1 (Figure 5B) and the disappearance and the regrowth of the cavity of organoid 2 (Figure 5C). The morphological sprouting started when the initial diameter of the organoid was around 50 μm (Figure 5D). From the results of volume monitoring of the three organoids grown for 7 days (Figure 5E), the initial appearance diameter of the organoids over 90 μm showed that their solid volume grew rapidly over time, as in organoids 1 and 2, while organoid 3, which had an initial appearance diameter near 50 μm, grew more slowly, increasing in volume only for the first 4 days, then remaining essentially unchanged.

**FIGURE 6:**
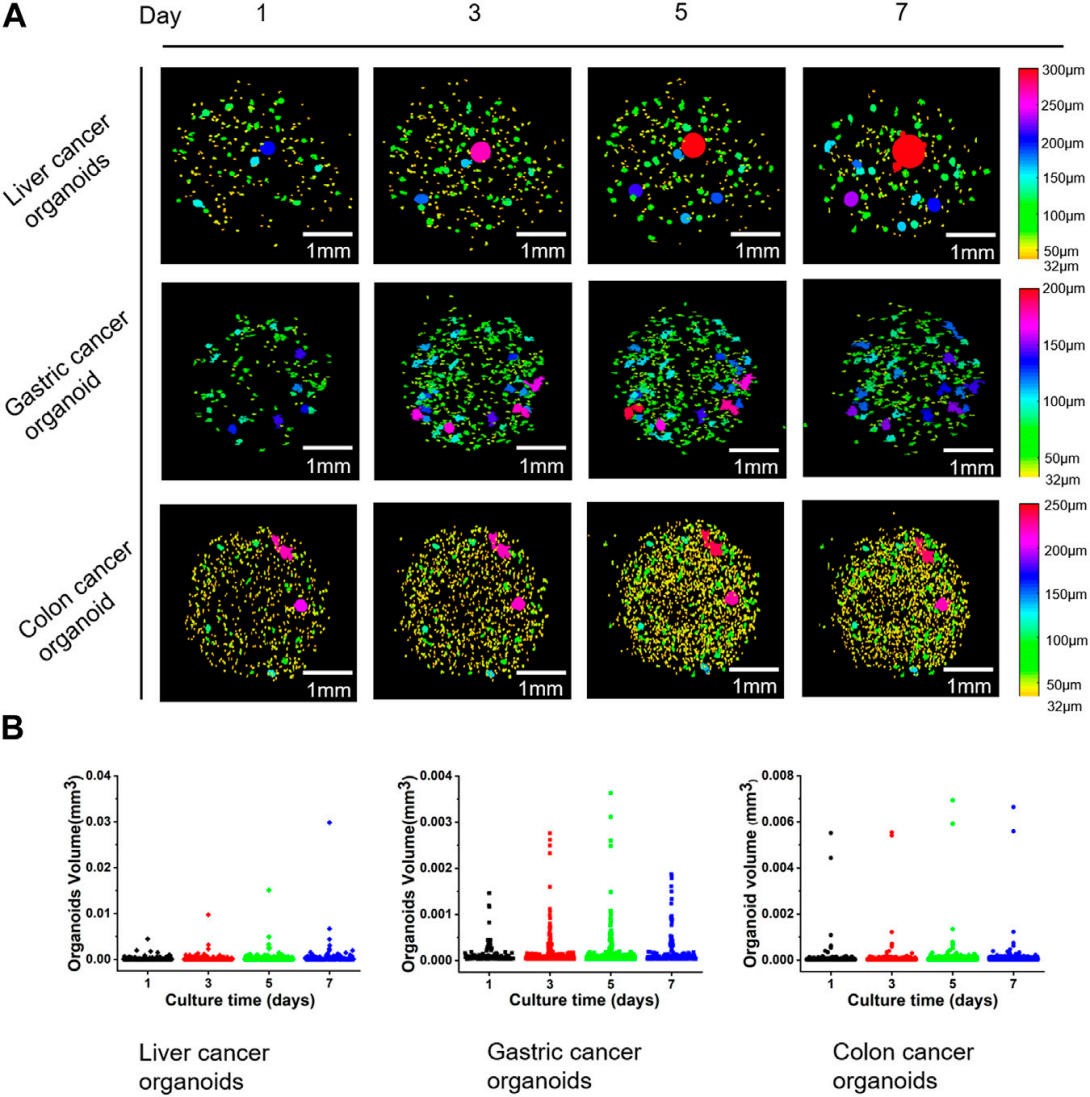
Examples of the changes of three different types of organoids over time and scatter plots of organoid volumes. **(A)** Three-dimensional reconstruction results of organoid volume changes over time for three types of liver, gastric, and colon cancers. **(B)** Scatter plots corresponding to the volume of the organoids in **(A)**.

Based on the accurate identification of the internal cavities of the organoids, we achieved a more accurate quantitative analysis of the organoids and monitored the changes in the number and volume of all organoids in the well plate over time. In order to visualize the growth and development of the organoids, we first filled the inside of the organoids and then calculated the equivalent diameter based on the volume of the organoids and assigned different colors to the organoids according to the diameter size. Figure 6 shows the scatter plots of growth status and volume changes of three groups of different types of organoid clusters at different culture time points. It should be pointed out that the organoids with diameter less than 32 μm did not visualized in Figure 6A to better display organoid clusters. According to the Figures 6A, B, it can be seen that on day 1 of culture, the percentage of liver and gastric cancer organoid clusters with diameters less than 50 μm exceeded 50%, while the percentage of intestinal cancer organoid clusters with diameters less than 50 μm exceeded 80%. During the subsequent growth process, the scale and volume of liver carcinoids showed significant growth, while the number did not change significantly, and the statistics showed that the percentage of organoids less than 50 μm in diameter continued to decrease; the diameter and number of gastric cancer organoids continued to increase, while the statistics showed that the percentage of organoids less than 50 μm in diameter increased slowly in the first 5 days and then decreased slightly; the diameter of intestinal cancer organoids grew slowly and the percentage of organoids with diameters less than 50 μm decreased significantly in the first 5 days and then increased slightly. In addition, the statistics of the last day showed that the percentage of the three types of organoids with diameters less than 50 μm was the smallest at 37.4%, indicating that a significant proportion of these organoids had no significant growth.

## 4 Discussion

Patient-derived cancer organoids (PCOs) are 3D *in vitro* miniaturized models that display spatial architecture strongly resembling the corresponding tumor tissues and recapitulate physiological functions of parent tissue, offering unprecedented opportunities for disease mechanism research, drug screening and personalized medicine. However, current detection methods for PCOs are usually destructive or provide only planar information, making it difficult to achieve long-term, non-destructive 3D monitoring and analysis of the PCOs cluster. In this study, we propose a OPO method for 3D imaging, segmentation and analysis of printed organoid clusters based on OCT imaging with deep CNNs, which can reconstruct the multiscale structure of organoid clusters within printed microbeads and visualize information about organoids, cavities and volumes.

The advantages of OCT, such as three-dimensional, non-invasive, and high-resolution imaging, allow it to monitor the growth and drug response of organoid clusters and capture the heterogeneity of PCOs. However, the sensitivity fall-off effect of OCT may generate signal variances in different depths of the organoid, and the low contrast and unavoidable speckle noise present in OCT images, which hamper the organoids identification. Previous studies (Nuciforo et al., 2018; Ngo et al., 2020) used conventional image processing to analyze organoid OCT images shows difficulties in segmenting organoids with scales smaller than 90 μm. However, the CNN model designed in this paper can extract the printed microbead matrix region of interest by a simple VGG-Unet (Figure 2A). On the other hand, the accuracy of organoid segmentation with diameters around 50 μm could be improved by the designed EGO-Net (Table 1). Moreover, the introduction of 3D information and ensured the continuity of segmentation (Figure 4) by unidirectional continuous 3-frame image input combined with two neural network predictions in the mean orthogonal direction information would be beneficial to reduce the deformation (Figure 4D3) and recognition deficit (Figure 4F3) of 3D organoid reconstruction. The CNN model designed in this paper exhibits good robustness and adaptability, providing satisfactory segmentation results for multi-scale organoid in OCT images with low contrast and much noise (Figure 3), showing excellent segmentation accuracy for small-scale organoid (d < 90 μm) and good prediction and identification readouts of organoid of different types of patient origin for liver, gastric and colon cancers with large morphological and density differences (Figure 6A).

Also, the CNN model we proposed in this paper is able to identify the edges of printed microbead matrix, which can be further used to calibrate the printing position. Moreover, the volume error of microbead printing would also be calibrated by quantifying the volume of the region, which is paramount for the automation of organoid preparation. The proposed organoid tracking analysis method in this paper enables the visualization of morphology and internal structure evolution of individual organoid at different time points (Figures 5B–D), and the 3D volume mapping helps to understand the underlying cellular dynamics, such as fusion, luminal dynamics, migration, and rotation, in more detail from different perspectives. The quantitative tracking analysis of individual organoid morphological parameters (Figure 5E) helps to explore the heterogeneity of PCOs for precise tumor drug use (Boehnke et al., 2016). Although the current organoid tracking analysis method has achieved the expected results, there are still limitations. The error of tracking is closely related to the segmentation accuracy, and the error of segmentation leads to the error of tracking. Large-sized organoids (d > 90 μm) are tracked accurately because they are easy to segment and errors are mainly concentrated at the edges. The segmentation accuracy of the model for the organoids with diameters between 50 and 90 μm is 83.9%. Although there is a small amount of organoid identification error, it can also be tracked accurately basically. For the organoids with diameter less than 50 μm, the identification accuracy of our segmentation algorithm is not high enough, and there is a situation that some organoid below 32 μm grows to about 50 μm, so the identification error of small-sized organoids with diameter less than 50 μm can easily lead to the error of tracking results. In this paper, we further demonstrated the changes of different types of patient-derived cancer organoid clusters over time (Figure 6A), and achieved quantification and analyze of the number and volume changes of organoid clusters (Figure 6B). It demonstrated that the growth rate of organoid was influenced by the initial scale which associates closely with the viability of organoid. In fact, the diameter and morphological characteristics of the organoid are key indicators of organoid maturation. Thus, the accurate assessment of the diameter and morphological characteristics would be beneficial to guide organoid culture. In addition, the accurate quantification and tracking of multiple scales of organoids helps to observe the growth and structural evolution of organoid clusters as well as to assess the appropriate developmental time scale (Yavitt et al., 2021), enhancing the understanding of early organoid formation mechanisms, and helping guide large-scale culture of the organoid. For example, in this paper, we found that an overall shrinking of the organoid (Figure 6A), known as size oscillations, occurs when the diameter of intestinal and gastric organoids grows to near 200 μm (Hof et al., 2021). In the next step, we consider combining classification algorithms to distinguish different morphological organoids and introducing OCT attenuation coefficients, dynamic OCT (Scholler et al., 2020; Yang et al., 2020; Ming et al., 2022) to further characterize the viability and motility of organoids and provide powerful tools for organoid growth, and drug screening based on structure-function imaging.

## 5 Conclusion

In this study, we propose a three-dimensional imaging, segmentation and analysis method of printed organoid clusters based on OCT with CNNs (OPO), which reconstruct the multi-scale structure of organoid clusters within printed microbeads and achieve tracking and quantitative analysis of organoids. By tracking individual organoid and quantifying morphological parameters such as number and volume of organoid by CNN prediction results, the growth, structural evolution and heterogeneity analysis of organoid clusters can be realized. The proposed method is valuable for the study of organoid growth, drug screening and tumor drug sensitivity detection based on organoids.

## Data Availability

The raw data supporting the conclusion of this article will be made available by the authors, without undue reservation.
